# ﻿Novelties in the genus *Ardisia* (Primulaceae) from Vietnam: a new species and two new records

**DOI:** 10.3897/phytokeys.260.154984

**Published:** 2025-07-16

**Authors:** Do Van Hai, Tran Duc Thien, Bui Hong Quang, Nguyen The Cuong, Gang Hao

**Affiliations:** 1 Department of Botany, Institute of Biology, Vietnam Academy of Science and Technology, 18 Hoang Quoc Viet, Cau Giay, Hanoi 10072, Vietnam Graduate University of Science and Technology Hanoi Vietnam; 2 Graduate University of Science and Technology, Vietnam Academy of Science and Technology, 18 Hoang Quoc Viet, Cau Giay, Hanoi 10072, Vietnam Vietnam Academy of Science and Technology Hanoi Vietnam; 3 College of Life Sciences, South China Agricultural University, Guangzhou 510642, China South China Agricultural University Guangzhou China

**Keywords:** Morphology, new species, Primrose family, subgenus *Bladhia*, *Tinus*, taxonomy

## Abstract

A new species, *Ardisiadahoaiensis* (Subgenus Tinus), is described from Lam Dong Province in the Central Highlands of Vietnam. Additionally, two taxa from the subgenus Bladhia of *Ardisia*: *A.purpureovillosa*, and *A.scalarinervis* are newly recorded from Vietnam. Photographic illustrations and voucher specimens for each species are provided.

## ﻿Introduction

The genus *Ardisia* Sw. (Primulaceae) is one of the largest genera in the subfamily Myrsinoideae of the expanded Primulaceae (APG 2016) and comprises approximately 740 species distributed primarily in the tropics, especially with high diversity in Southeast Asia, the Americas, Australia, and the Pacific (Chen and Pipoly 1996; Utteridge 2021; POWO 2025). Traditionally, *Ardisia* was classified within the Myrsinaceae by several taxonomists, including Chen and Pipoly (1996), Larsen and Hu (1996), Lien (2002), Hu and Vidal (2004). The genus is morphologically classified into 17 subgenera by Mez (1902), Stone (1993), Larsen and Hu (1996), Yang and Hu (2022). In Vietnam, eight sections (subgenera) are presented, and 101 species of *Ardisia* have been recorded, with several new species described after the revision completed by Lien (2002) in “Flora of Vietnam”. Notably new species include *Ardisiabanaensis* C.M. Hu & L. K. Phan; *A.sadirioides* C.M. Hu & L.K. Phan (Phan et al. 2013); *A.phankelociana* C.M. Hu & G. Hao (Hu et al. 2017); *A.daklakensis* C.M. Hu & Nuraliev (Nuraliev et al. 2020); and *A.patentiradiosa* C.M. Hu & Nuraliev (Nuraliev et al. 2021).

During our fieldwork in Dien Bien and Lai Chau provinces, northern Vietnam, we collected several *Ardisia* species, two of which did not match any known taxon in Vietnam. Specimens of these two species belong to the subgenus Bladhia (Thunb.) Mez, characterized by leaves that are sub-opposite to sub-verticillate or crowded towards the distal part or apex of the stem or branches, with margins densely serrulate to denticulate having small to larger teeth evenly arranged along the entire length of the margin. After examining the voucher specimens and type materials and consulting relevant literature (Chen and Pipoly 1996; Hu and Vidal 1996, 1997, 2004 ; Lien 2002), we concluded that the species from the two provinces above are *A.purpureovillosa*, and *A.scalarinervis*, representing new records for the flora of Vietnam.

During the same period, another plant species was collected from Lam Dong Province in the Central Highlands of Vietnam. This species has medicinal value and is being experimentally cultivated at the Central Highlands Research Center for Medicinal Materials (Lam Dong province). However, its scientific name remains controversial and difficult to determine; some scientists believed it to be *Ardisiasylvestris* Pit. Hence, the specimens were sent to the herbarium of the Institute of Ecology and Biological Resources, Vietnam Academy of Science and Technology at Hanoi (HN) for authentication of these materials. At the same time, the corresponding author (Gang Hao) visited HN and studied the plant materials with other authors. A critical review of the specimens led us to conclude that the specimens belong to the subgenus Tinus Mez, characterized by inflorescences axillary or more usually lateral arising directly from the branch or in the axils of fallen leaves (usually without a subtending leaf at anthesis); calyx lobes distinctly imbricate at anthesis, leaf margins entire, rarely finely to obscurely dentate. After recollecting the specimens together and comparing them with the previous specimens, as well as consulting literature (Chen and Pipoly 1996; Hu and Vidal 1996, 1997, 2004; Lien 2002), we concluded that this taxon represents a new species, which is described and illustrated below as *Ardisiadahoaiensis*.

## ﻿Materials and methods

The morphological description of the new species and records is based on the examination of both fresh and pressed specimens. Voucher specimens were prepared following the standard protocols (Davies et al. 2023). These specimens are deposited at the herbarium of the Institute of Biology, Vietnam Academy of Science and Technology (HN), and the herbarium of South China Botanical Garden, Chinese Academy of Sciences (**IBSC**). In addition, specimen images from Global Plants JSTOR (https://plants.jstor.org/) at the Kew Herbarium Catalogue (https://apps.kew.org/herbcat/gotoHomePage.do) and Plants of the World Online (POWO: https://www.plantsoftheworldonline.org/) were consulted. All measurements for the floral description were taken from herbarium specimens. Flowering and fruiting materials are indicated by ‘fl.’and ‘fr.’, respectively. Relevant taxonomic literature (e.g. Larsen and Hu 1991; Chen and Pipoly 1996; Hu and Vidal 1996, 1997, 2004; Lien 2002) was also consulted. The conservation assessments of these species were undertaken using IUCN Standards and Petitions Committee (2024).

## ﻿Taxonomic treatment

### ﻿ Ardisia (Tinus) dahoaiensis

Taxon classificationPlantaeEricalesPrimulaceae

D.V.Hai & G.Hao sp. nov.

47317BC7-CB17-5E12-AA0E-58350CC8A95C

urn:lsid:ipni.org:names:77365657-1

Figs 1 , 2

#### Type.

Vietnam • Lam Dong Province: Cat Tien District, Tien Hoang Commune; 11°40'27.2"N, 107°22'23.4"E, 325 m alt., 8 October 2024 (fl. & fr.); *Tran Trong Hung et al., DVH 448* (holotype HN!; isotypes HN!).

#### Diagnosis.

*Ardisiadahoaiensis* is the only species under this genus with the following combination of characters: leaves 6–12, alternate or forming a rosette, mainly clustered at the ends of branches, lamina subcoriaceous; margin entire or crenate in the upper part, with pustule-like structures along the crenations from projecting venation; inflorescence grows in extra axillary; flowers 5–7 merous; ovules ca. 16–18 arranged around on placenta.

#### Description.

Shrubs, 40–60 cm high, stem erect, usually not branched; young shoots villous, young stem pubescent; old stem smooth; base diameter ca. 5–7 mm. Leaves 6–12, alternate or forming a pseudo-verticillate, mainly clustered at the ends of branches; petioles 10–15 mm long, winged by the decurrent leaf base, covered with dense, white simple hairs; lamina subcoriaceous, with sparse black gland dots throughout abaxially, raised between the venation; leaf blade broadly elliptic or oblanceolate-obovate, 25–35 × 8–13 cm; young leaves purple, especially the veins, mature leaves dark green above, pale green beneath; base attenuate; margin entire or crenate in the upper part, with pustule-like structures along the crenations from projecting venation; apex acute, adaxial surface glabrous, abaxial surface copiously rusty villose especially on the midrib, and with many glandular dots, visible under hand lens; mid-rib flat above, raised below; lateral veins 12–18 pairs, irregularly spaced, with short intersecondary veins, slender, distinct but shorter than lateral veins, angle to the midrib about 45°−50°, arcuate upward and joining at the marginal vein, distinct on the adaxial surface, prominent on abaxial side; reticulation of veins visible. Inflorescence grows in extra axillary, 4–6 cm long, usually unbranched, 12–16-flowered at the top, condensed racemose, forming short clusters or umbels; bracts foliaceous, linear to lanceolate, 3.5–3.8 × 0.6–0.7 cm, subsessile, apex acuminate, densely pubescent on both sides, margin revolute. Flowers 5–7 merous; pedicels purplish-red, 1.6–2 cm long, slender, puberulent. Calyx 5-(−7) lobed, 2.2–2.5 × 1.3–1.5 mm long, purplish-red, split to near base; lobes oblong-ovate, acute at apex; orange-pink punctate, margin minutely ciliolate, pubescent abaxially, glabrous adaxially. Corolla tube ca. 1.3–1.5 mm long, lobes 5-(−7), white to light pink except the pinkish along the mid-petal and pinkish base, covered with dense, brown gland dots, lobes broadly ovate, 6–6.5 × 4–4.5 mm, glabrous on both surfaces, apex acute and tips slightly folded inwards; stamens 5-(−7), yellowish, subsessile, anthers narrowly ovate-lanceolate, longer than filaments, 4–4.5 × 1.5 mm, apiculate, glabrous throughout, gland dotted abaxially, thecae not locellate, dehiscent by longitudinal slits. Ovary superior, ovoid, 1.5–1.6 × 1.3–1.4 mm, glabrous, gland dotted yellow; ovules 16–18 arranged around on placenta; style protruding beyond stamens, 2.5–3 mm long, irregularly curved and narrow to the apex, stigma minute. Fruit drupaceous, globose, 5 mm in diameter, young fruits green, glabrous, with gland dotted yellow, mature fruits red; pistil exists at the apex; exocarp thin, mesocarp white and soft, endocarp red and hard, has many longitudinally ribbed. Seed smooth, with holes at the base; endosperm white, thick, horizontal.

#### Distribution and habitat.

Endemic to Vietnam, Lam Dong Province (Da Hoai district); currently known only from the type locality. It grows under the shade of evergreen broad-leaved forest on soils and stone, at an elevation of 300–400 m alt.

#### Phenology.

Flowering and fruiting from September to October.

#### Etymology.

The specific epithet of the new species refers to Da Hoai district (Lam Dong Province) in the Central Highlands of Vietnam, where it was first collected.

#### Vernacular name.

Khôi tía.

#### Preliminary conservation status.

This new species has been collected from three localities in Da Hoai district, Lam Dong province. It is quite common, but forest habitats can be impacted by humans due to farming or infrastructure constructions, sometimes harvested in small amounts for medicinal use. Further surveys are needed to understand the threats at the type locality and if the species is distributed outside the current area, and until these data are obtained, the species is assessed as Data Deficient (DD) (IUCN Standards and Petitions Committee 2024).

#### Additional specimens examined

**(paratypes).** VIETNAM, Lam Dong Province • Da Hoai District, Ha Lam commune, 17 September 2023, *Tran Hung et al. DVH 17092023-1* (HN, IBSC) • Trieu Hai Commune, 17 September 2023, *Tran Hung et al. DVH 17092023-2* (HN, IBSC); (HN, IBSC) • Tien Hoang commune, 17 September 2023, *Tran Hung et al. DVH 17092023-3* (HN, IBSC).

#### Notes.

**Figure 1.**
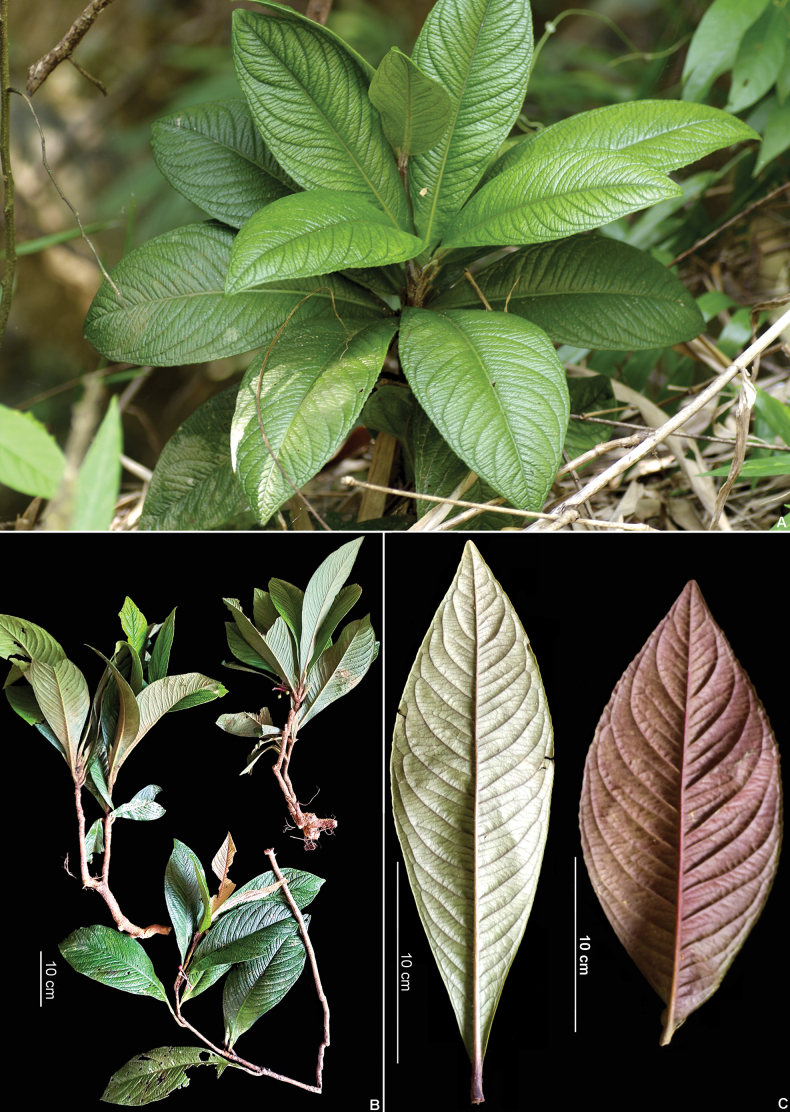
*Ardisiadahoaiensis*. **A.** Habitat; **B.** Habit; **C.** Leaves, adaxial and abaxial sides. Photos by N. T. Hoa.

**Figure 2.**
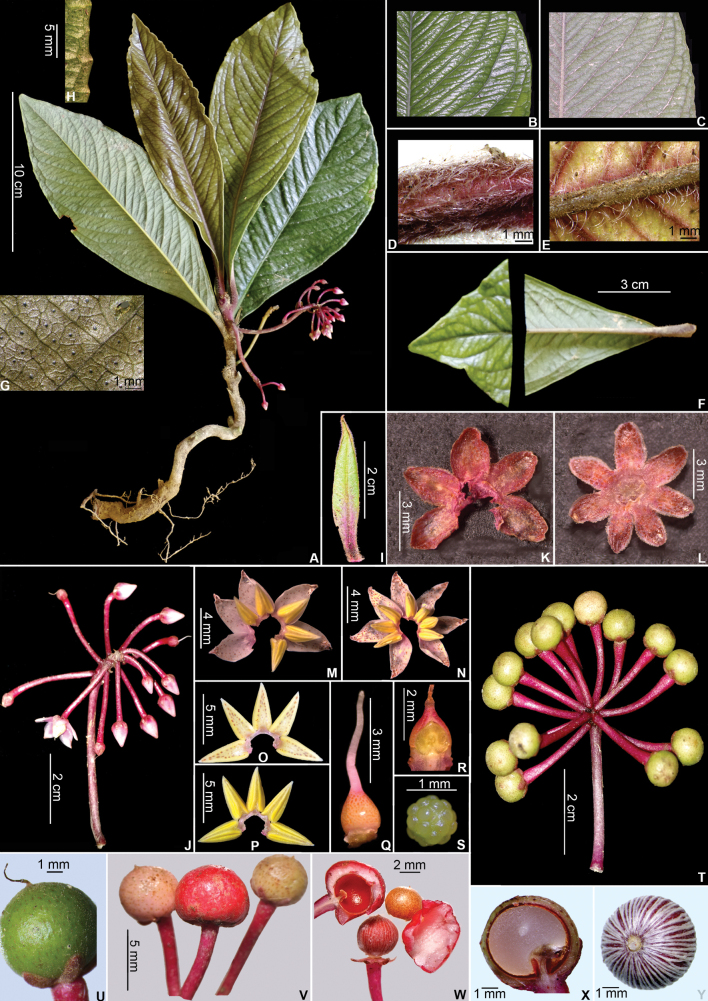
*Ardisiadahoaiensis*. **A.** Habit; **B.** Leaf blade margin, adaxial view; **C.** Leaf blade margin, abaxial view; **D.** Hairs on petiole; **E.** Hairs on midvein (abaxial) **F.** Leaf apex and base; **G.** Glandular dots on abaxial surface; **H.** Pustule-like structures along the crenation; **I.** Bract; **J.** Inflorescence; **K.** Calyx, adaxial side; **L.** Heptamerous calyx, adaxial side; **M.** Corolla opened, showing stamens; **N.** Heptamerous flower and stamens, adaxial side; **O.** Androecium, abaxial side; **P.** Androecium, adaxial side; **Q.** Gynoecium; **R.** Ovary, longitudinal section; **S.** Placenta; **T.** Infructescence; **U–W.** Fruit; **X.** Fruit, longitudinal section; **Y.** Seed. Photos by N. T. Hoa & V. A. Thuong.

The exact placement of the subgenus of the new species is not easy, and sometimes confusing. At first we thought it belonged to the subgen. Akomos, which is characterized by inflorescences that are always terminal or both terminal and axillary, on branches with spirally arranged leaves, with inflorescences at the distal portion of branches often without subtending leaves and distinctly pedunculate; leaf margin usually entire, sometimes dentate in the upper part; and the plants drying brown to chocolate-brown; calyx with lobes spread out at anthesis, little or not imbricate, plants generally scaly (Hu and Vidal 2004). However, after a detailed examination of the specimens and descriptions of the species, we found that the described species has the following characteristics: the sepals are overlapping (Fig. 2J, K), and young stem pubescent; old stem smooth; the lateral inflorescences arising directly from the main stem (Fig. 2A), serrulate teeth toward the distal part of the margin (Fig. 1C). These features are consistent with the subgen. Tinus Mez (Hu and Vidal 2004; Utteridge et al. 2023).

Under Ardisiasubgen.Tinus, Vietnam has 14 species (Hu and Vidal 2004; Lien 2002, 18 speices in Thailand (Larsen and Hu 1996). This new species is distinct from all known species of the subgen. Tinus. Among them, the new species has the most similar characteristics to the two species *A.albomaculata* and *A.brunnescens* (subgen. Akomos). In addition to the characteristics of different subgenera such as indumentum, sepals, inflorescens, it differs from *A.albomaculata* in the leaf texture, venation and position of inflorescence (i.e., subcoriaceous; lateral veins 12–18 pairs, irregularly spaced, with short intersecondary veins, slender, distinct but shorter than lateral veins, angle to the midrib about 45°-50°; inflorescence extra axillary, condensed racemose). It differs from *A.brunnescens* by the indumentum on leaves, calyx (i.e., adaxial surface glabrous, abaxial surface copiously rusty villose especially on the midrib, and with many glandular dots). The detailed comparison between *A.dahoaiensis*, *A.albomaculata* and *A.brunnescens* is given in Table 1.

**Table 1.** Morphological comparison of *Ardisiadahoaiensis*, *A.albomaculata* (data from Pitard 1930; Chen and Pipoly 1996; Hu and Vidal 2004 and from our study of the type specimen P00075000, image https://plants.jstor.org/stable/10.5555/al.ap.specimen.p00075000) and *A.brunnescens* (data from Walker 1937 and from our study of the type specimen NY00016398, image https://plants.jstor.org/stable/10.5555/al.ap.specimen.ny00016398).

Morphological characters	*A.dahoaiensis*	*A.albomaculata*	*A.brunnescens*
Habit	shrubs 40–60 cm tall	shrubs 50 cm tall	shrubs 0.5–1(–3) m tall
Petiole length	10–15 mm long, winged by the decurrent leaf base, covered with dense, white simple hairs	7–10 mm long	7–12 mm long, marginate, glabrescent
Lamina	6–12, broadly elliptic or oblanceolate-obovate	3-4, broadly oblong or obovate	many, elliptic
Leaf size	25–35 × 8–13 cm	22–24 × 7–9 cm	8–14 × 3.5–6 cm
Leaf texture	subcoriaceous	chartaceous	chartaceous
Leaf apex	acute	acuminate, obtuse or mucronulate	acute or broadly acuminate
Lateral veins	12–18 pairs, distinct on the adaxial surface, prominent on abaxial side	15–18 pairs, obliquely ascending, fine, clearer below, forming with the tertiary venation a network with numerous and very small meshes, equally prominent on both faces	10–15 on each side of midrib
Inflorescences	extra axillary, 4–6 cm long, usually unbranched, 12–16 flowered at the top, condensed racemose, forming short clusters or umbels	axillary, 4–6 cm long at the fruiting stage; axis 3–5 cm long, unbranched, bearing at the top 12–15 flowers grouped in an umbel; fruiting pedicel 12–15 mm long	subterminal, paniculate, on specialized lateral branches 5–9 cm, with 1 or 2 reduced apical leaves, branches umbellate
Pedicels	16–20 mm long	12–15 mm long	ca. 10 mm long
Calyx lobes	oblong-ovate, 2.2–2.5 mm long, orange-pink punctate	oblong, ca. 2.5 mm long, punctate	ovate, ca. 1.5 mm long, punctate
Fruits	globose, 5 mm in diameter, gland dotted yellow	subglobose, 6 × 5.5 mm, gland dotted black	globose, 6–7 mm in diameter, pellucid punctate
Flowering and fruiting	September to October	Fruiting in March	Flowering April, fruiting October to January (next year)

The new species has an unusual feature in that, besides the usual pentamerous flowers with five sepals and corolla lobes and five stamens, hexamerous-heptamerous flowers were frequently observed, possessing six to seven sepals, corolla lobes and stamens. This feature is different from *A.albomaculata* (5-merous flowers). Furthermore, the population of this new species is quite close geographically to the *A.albomaculata*, distributed in Dong Nai province. However, the new species also has a different fruiting season compared to *A.albomaculata* (March). In addition, *A.albomaculata* also has the distinct characteristic of having scaly sepals (as observed from the type specimens [P00075000, P00087802] placing it in subgen. Akomos [Hu and Vidal 2004]). Taken together with the morphological differences from known species, it is apparent that the newly collected material indeed represents a species new to science, which we compared in detail to *A.albomaculata* and *A.brunnescens* and described here as *Ardisiadahoaiensis* D.V. Hai & G. Hao.

##### ﻿New records for Vietnam

### ﻿ Ardisia (Bladhia) purpureovillosa

Taxon classificationPlantaeEricalesPrimulaceae

C.Y.Wu & C.Chen ex C.M.Hu, Acta Bot. Austro Sin. 6: 29 (1990).

2041B84F-DB4D-503E-B878-7AF0A07075EC

Fig. 3

#### Type.

China • Yunnan Province: Malipo, Laojunshan, in broad-leaf forest, 19 May 1962, *K.M. Feng 22682* (holotype: IBSC!).

#### Specimens examined in Vietnam.

Lai Chau Province, Muong Te District, Mu Ca Commune, in evergreen forest, 433 m alt., 22°33'56.9"N, 102°27'20.6"E, 9 April 2022, *Do Van Hai et al. ĐTCS 169* [fl.] (HN); Ka Lang commune, 23 March 2025, *Do Van Hai et al. ĐLTB 594* [fl.] (HN).

**Figure 3.**
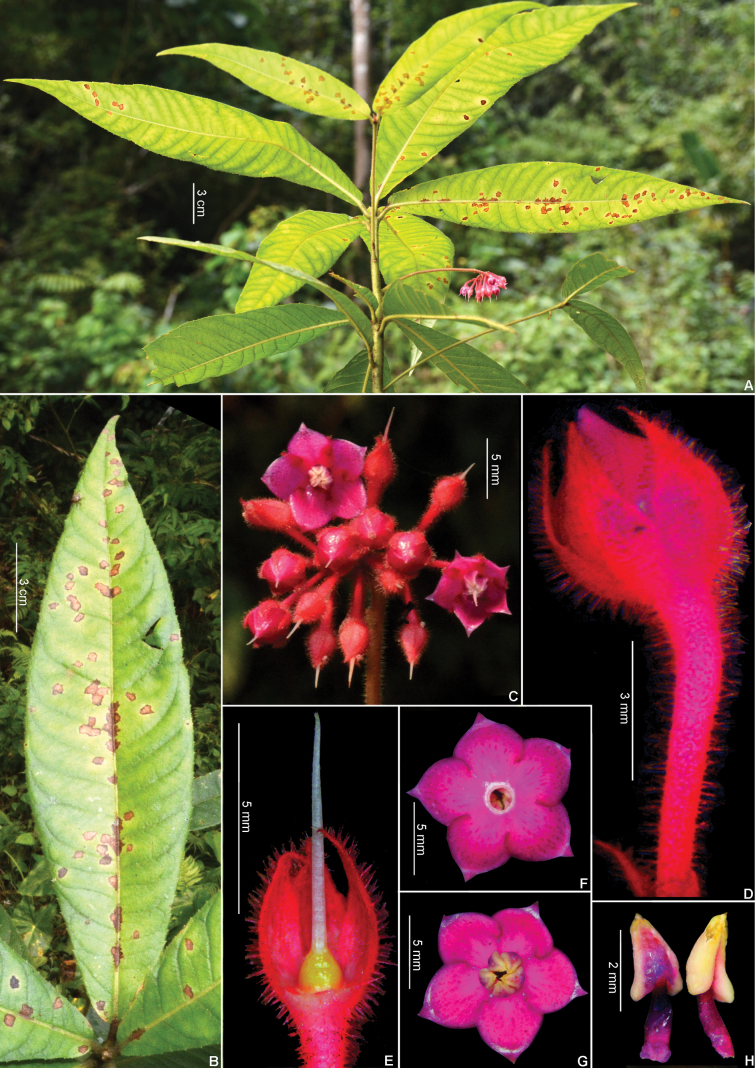
*Ardisiapurpureovillosa* C.Y.Wu & C.Chen ex C.M.Hu. **A.** Flowering branch; **B.** Leaf blade, adaxial view; **C.** Inflorescence; **D.** Flower bud; **E.** A flower after moving corolla and stamens, showing calyx and pistil; **F.** Corolla, abaxial side; **G.** Corolla opened, showing stamens; **H.** Stamens. Photos by T. T. Bach & D. V. Hai.

#### Distribution.

China (Guangxi, Hainan, Yunnan) and Vietnam (Lai Chau).

#### Ecology in Vietnam.

During the field survey, two populations were was found at an altitude of approximately 433 m; in evergreen forest, moist places.

#### Phenology.

Flowering from April to May, fruiting from September to November in China (Chen and Pipoly 1996). Flowering specimens were collected from Vietnam during March to April.

#### Preliminary conservation status.

In Vietnam, the newly recorded species was collected from two populations in Muong Te district, Lai Chau Province. These populations are distributed within protected forests, but there is no data on population assessments in the field. Based on our predictions, the species could occur in other locations with similar habitat conditions. Therefore, it may be classified as ‘Data Deficient’ (DD) according to the IUCN Red List Categories and Criteria (IUCN 2024).

### ﻿ Ardisia (Bladhia) scalarinervis

Taxon classificationPlantaeEricalesPrimulaceae

E.Walker, J.Washington Acad. Sci. 21: 477 (1931).

3A3B376E-9078-59DF-B341-C4D7E69C9F8A

Fig. 4

#### Type.

China • Yunnan Province: Szemao, 4500 feet alt., *A. Henry 12021* (holotype: US! [US00116206]; isotypes: A! [A00025264], K! [K000756647]).

**Figure 4.**
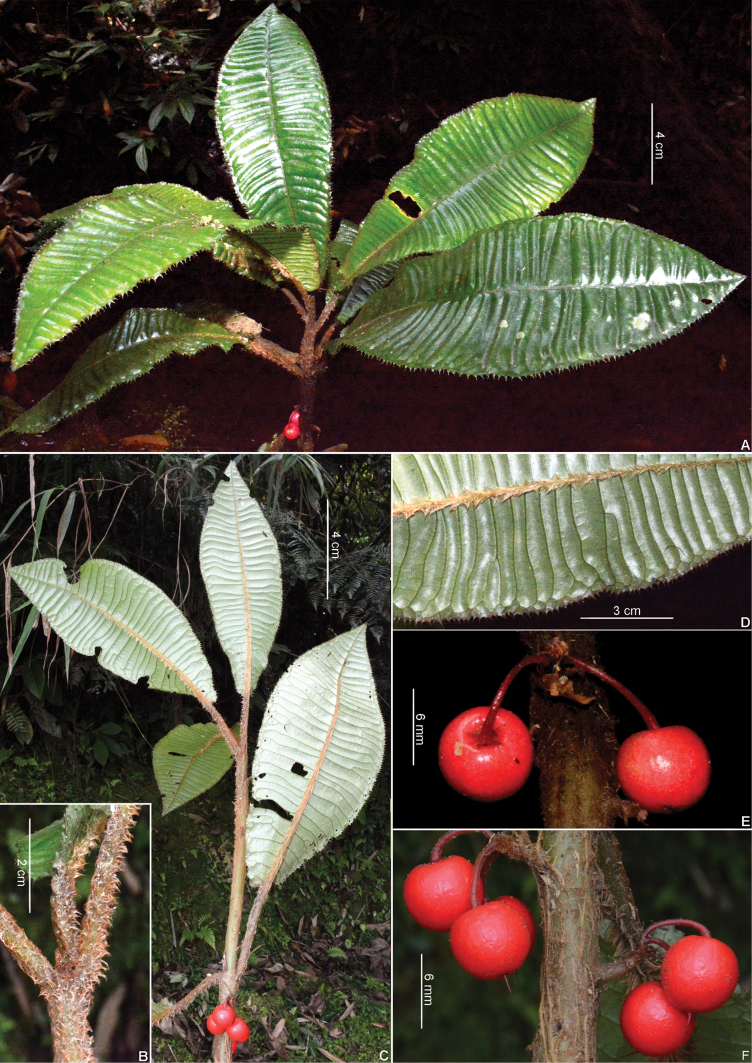
*Ardisiascalarinervis* E.Walker. **A.** Habit; **B.** Stem and petiole; **C.** Branch with leaves on the abaxial view and fruits; **D.** Leaf blade margin, abaxial view; **E, F.** Fruits. Photos by D. V. Hai.

#### Specimens examined in Vietnam.

Dien Bien Province, Muong Nhe District, Sin Thau Commune, in evergreen forest: 770 m alt., 22°23'59.2"N, 102°14'12.4"E, 12 April 2022, *Do Van Hai et al. CSCL 88* [fr.] (HN); Lai Chau Province, Muong Te District, Mu Ca Commune, in evergreen forest: 621 m alt., 22°29'45.7"N, 102°26'01.1"E, 8 April 2022, *Do Van Hai et al. ĐTCS 98* [fr.] (HN); ibid., 22 March 2025, *Do Van Hai et al. ĐLTB* 592 [fr.] (HN).

#### Distribution.

China (Yunnan) and Vietnam (Dien Bien, Lai Chau).

#### Ecology in Vietnam.

In the field survey, only two populations were found at an altitude of 621 m and 770 m, respectively; in evergreen forest, moist places.

#### Phenology.

Fruiting from February to April in China (Chen and Pipoly 1996). Fruiting from March to April in Vietnam.

#### Preliminary conservation status.

*Ardisiascalarinervis* was previously known as an endemic species to China and distributed in Yunnan. Although only two populations were collected in Muong Te and Muong Nhe districts in Vietnam, more individuals are expected to occur there and the habitat is currently well-protected from anthropogenic activities under the law. Thus, it is appropriate to place this species under the category Least Concern (LC) following IUCN Red List (IUCN 2024).

## Supplementary Material

XML Treatment for Ardisia (Tinus) dahoaiensis

XML Treatment for Ardisia (Bladhia) purpureovillosa

XML Treatment for Ardisia (Bladhia) scalarinervis

## ﻿Additional information

### Use of AI

No use of AI was reported.

### Author ORCIDs

Do Van Hai, https://orcid.org/0000-0001-7841-0585

Tran Duc Thien https://orcid.org/0000-0001-9635-7060

Bui Hong Quang https://orcid.org/0000-0001-6878-7514

Nguyen The Cuong https://orcid.org/0000-0001-5159-3844

Gang Hao http://orcid.org/0000-0001-6125-9305

### Data availability

All of the data that support the findings of this study are available in the main text.
